# Supplementary material for: Effects of similarity networks in graph-based multi-omics classification
Source: PLoS One. 2026 Mar 19;21(3):e0344754. doi: 10.1371/journal.pone.0344754 (PMC13001923; doi:10.1371/journal.pone.0344754)
Supplement: S1 Fig — (A) Radar plot illustrating comparative metric distributions across similarity methods. (B) Violin plot showing the spread and central tendency of classification metrics. (PDF) [file pone.0344754.s001.pdf]

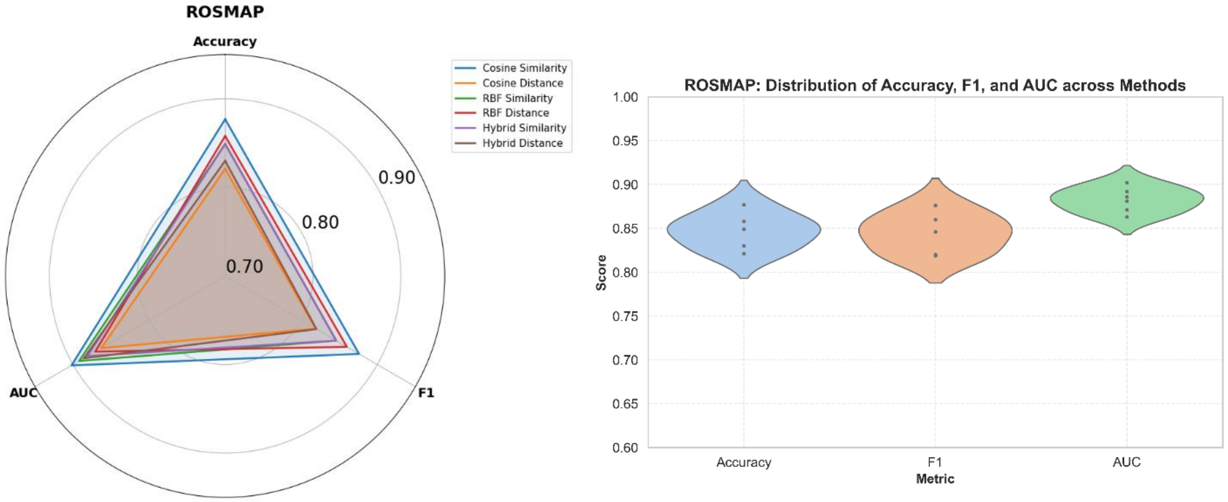


**S1 Fig. Performance metric visualizations for the ROSMAP dataset across similarity network variants.** (A) Radar plot illustrating comparative metric distributions across similarity methods. (B) Violin plot showing the spread and central tendency of classification metrics.
