# Supplementary material for: Effects of similarity networks in graph-based multi-omics classification
Source: PLoS One. 2026 Mar 19;21(3):e0344754. doi: 10.1371/journal.pone.0344754 (PMC13001923; doi:10.1371/journal.pone.0344754)
Supplement: S2 Fig — (A) Radar plot illustrating comparative metric distributions across similarity methods. (B) Violin plot showing the spread and central tendency of classification metrics. (PDF) [file pone.0344754.s002.pdf]

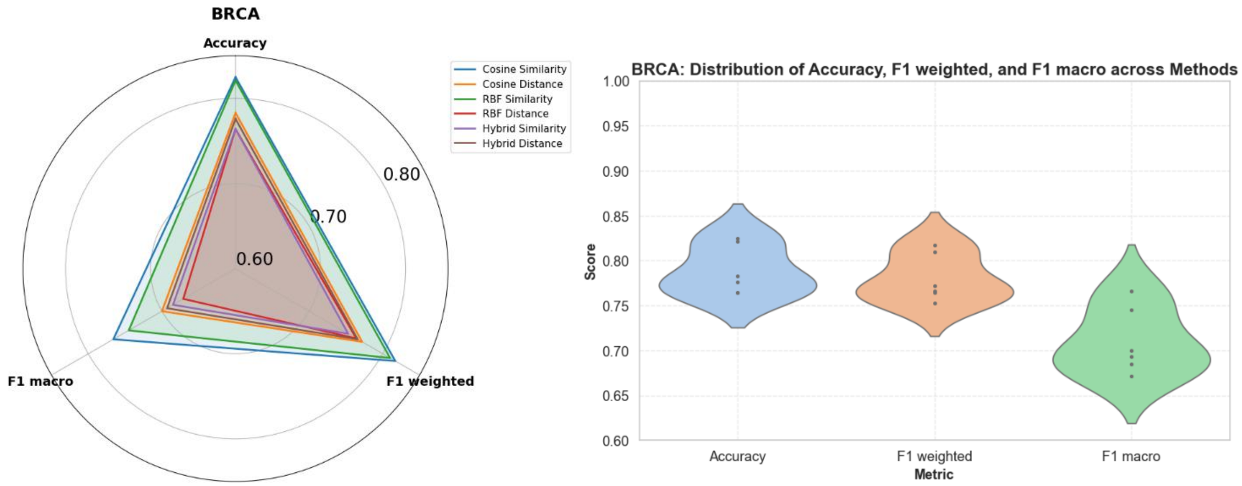

S2 Fig. Performance metric visualizations for the BRCA dataset across similarity network variants. (A) Radar plot illustrating comparative metric distributions across similarity methods. (B) Violin plot showing the spread and central tendency of classification metrics.
