# Supplementary material for: Effects of similarity networks in graph-based multi-omics classification
Source: PLoS One. 2026 Mar 19;21(3):e0344754. doi: 10.1371/journal.pone.0344754 (PMC13001923; doi:10.1371/journal.pone.0344754)
Supplement: S3 Fig — Line plot illustrating AUC variance trends across similarity metrics. (PDF) [file pone.0344754.s003.pdf]

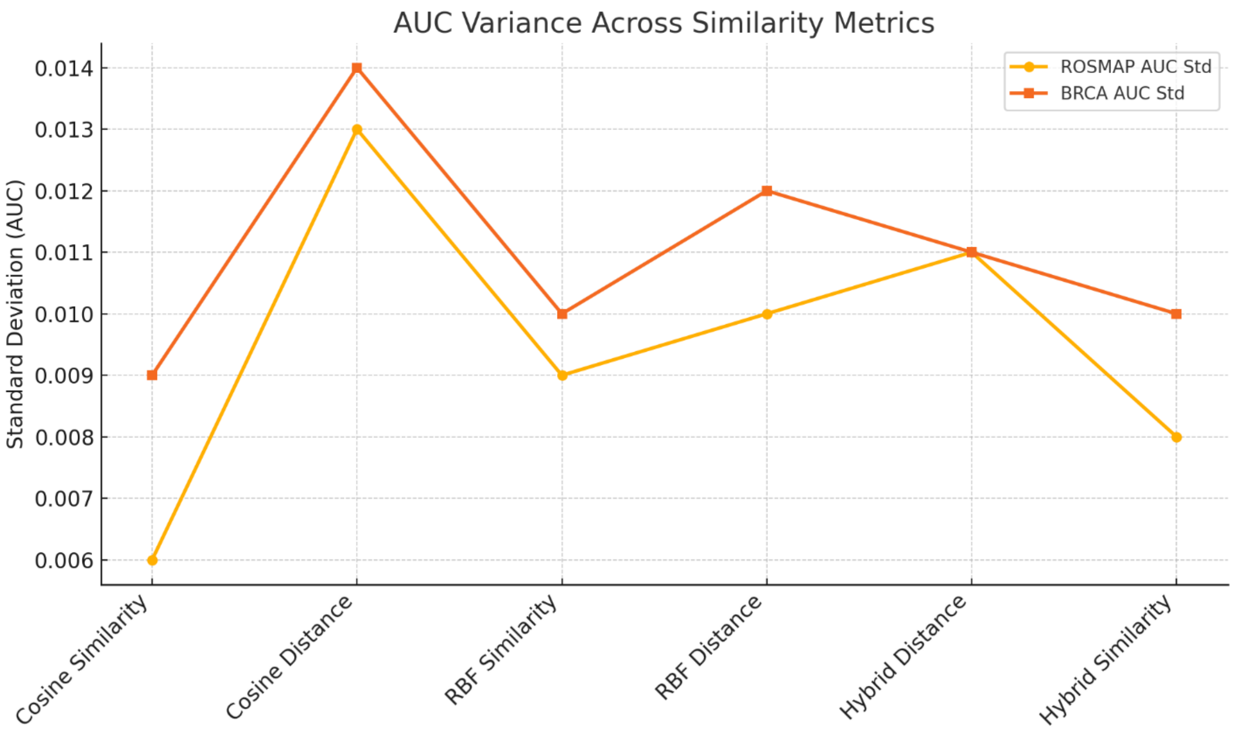

S3 Fig. AUC variability across similarity networks for BRCA and ROSMAP datasets. Line plot illustrating AUC variance trends across similarity metrics.
