# Supplementary material for: Effects of similarity networks in graph-based multi-omics classification
Source: PLoS One. 2026 Mar 19;21(3):e0344754. doi: 10.1371/journal.pone.0344754 (PMC13001923; doi:10.1371/journal.pone.0344754)
Supplement: S1 Table — (PDF) [file pone.0344754.s004.pdf]

S1 Table. Dataset composition and selected features used for training in BRCA and ROSMAP. Modalities: mRNA / DNA methylation / miRNA.

Dataset	Classes	Raw Features	Selected Features
BRCA	5 (PAM50)	20,531 / 20,106 / 503	1000 / 1000 / 503
ROSMAP	2 (AD vs. NC)	55,889 / 23,788 / 309	200 / 200 / 200
