# Supplementary material for: Effects of similarity networks in graph-based multi-omics classification
Source: PLoS One. 2026 Mar 19;21(3):e0344754. doi: 10.1371/journal.pone.0344754 (PMC13001923; doi:10.1371/journal.pone.0344754)
Supplement: S2 Table — (PDF) [file pone.0344754.s005.pdf]

S2 Table. Classification performance metrics (Accuracy, F1-score, AUC) for each similarity method on BRCA and ROSMAP datasets.

Similarity Metric	ROSMAP			BRCA		
	ACC	F1	AUC	ACC	F1 weighted	F1 macro
Cosine-sim	0.877	0.876	0.902	0.825	0.817	0.766
Cosine-dist	0.821	0.819	0.869	0.783	0.772	0.700
RBF-sim	0.849	0.846	0.892	0.821	0.810	0.745
RBF-dist	0.858	0.860	0.871	0.764	0.746	0.671
Hybrid-dist	0.830	0.820	0.886	0.776	0.766	0.693
Hybrid-sim	0.849	0.846	0.881	0.764	0.753	0.685
