# Supplementary material for: Effects of similarity networks in graph-based multi-omics classification
Source: PLoS One. 2026 Mar 19;21(3):e0344754. doi: 10.1371/journal.pone.0344754 (PMC13001923; doi:10.1371/journal.pone.0344754)
Supplement: S3 Table — (PDF) [file pone.0344754.s006.pdf]

S3 Table. Standard deviation of AUC across five randomized splits for each similarity network. Lower values indicate more consistent performance.

Similarity Metric	ROSMAP AUC Std	BRCA AUC Std
Cosine Similarity	0.006	0.009
Cosine Distance	0.013	0.014
RBF Similarity	0.009	0.010
RBF Distance	0.010	0.012
Hybrid Distance	0.011	0.011
Hybrid Similarity	0.008	0.010
