# Supplementary material for: Effects of similarity networks in graph-based multi-omics classification
Source: PLoS One. 2026 Mar 19;21(3):e0344754. doi: 10.1371/journal.pone.0344754 (PMC13001923; doi:10.1371/journal.pone.0344754)
Supplement: S4 Table — (PDF) [file pone.0344754.s007.pdf]

S4 Table. Performance Comparison of Similarity Metrics for ROSMAP Binary Classification.

Similarity Metric	95% CI (AUC)	Cohen's d
Cosine Similarity	[0.897, 0.907]	–
Cosine Distance	[0.858, 0.880]	3.30 (Huge)
RBF Similarity	[0.884, 0.900]	1.30 (Large)
RBF Distance	[0.862, 0.880]	3.75 (Huge)
Hybrid Distance	[0.876, 0.896]	1.81 (Large)
Hybrid Similarity	[0.874, 0.888]	2.97 (Huge)

*Note: To supplement the paired t-tests, we calculated 95% Confidence Intervals (CI) and Cohen's d effect sizes for the AUC and F1-score metrics across all six similarity construction strategies.
