# Supplementary material for: Effects of similarity networks in graph-based multi-omics classification
Source: PLoS One. 2026 Mar 19;21(3):e0344754. doi: 10.1371/journal.pone.0344754 (PMC13001923; doi:10.1371/journal.pone.0344754)
Supplement: S5 Table — (PDF) [file pone.0344754.s008.pdf]

S5 Table. Performance Comparison of Similarity Metrics for BRCA Multiclass Classification.

Similarity Metric	95% CI (AUC)	Cohen's d
Cosine Similarity	[0.809, 0.825]	–
Cosine Distance	[0.760, 0.784]	3.84 (Huge)
RBF Similarity	[0.801, 0.819]	0.74 (Med-Large)
RBF Distance	[0.735, 0.757]	6.67 (Huge)
Hybrid Distance	[0.756, 0.776]	5.06 (Huge)
Hybrid Similarity	[0.744, 0.762]	6.72 (Huge)

*Note: F1-weighted used as the primary performance proxy for BRCA multiclass comparison.
*Note: To supplement the paired t-tests, we calculated 95% Confidence Intervals (CI) and Cohen's d effect sizes for the AUC and F1-score metrics across all six similarity construction strategies.
