# Supplementary material for: Effects of similarity networks in graph-based multi-omics classification
Source: PLoS One. 2026 Mar 19;21(3):e0344754. doi: 10.1371/journal.pone.0344754 (PMC13001923; doi:10.1371/journal.pone.0344754)
Supplement: Attachment — Submitted filename: Response to Reviewers.pdf [file pone.0344754.s009.pdf]

Reviewer Comments

Reviewer #1:

All corrections made in response to comments raised by Reviewer 1 are highlighted in yellow in the revised manuscript.

1. The manuscript is well-structured but requires deeper justification for selecting only BRCA and ROSMAP datasets to validate generalizability.

   ## Response:

   The authors thank the reviewer for this constructive suggestion. We selected the BRCA and ROSMAP datasets as gold-standard benchmarks because they represent fundamentally different disease pathologies acute neoplastic heterogeneity versus chronic neurodegeneration allowing us to validate our model across both multiclass and binary classification tasks. These datasets provide high-quality, matched multi-omics profiles (mRNA, DNA methylation, and miRNA) essential for stable Graph Convolutional Network training. To address this point, we have added a paragraph to the "Dataset Description" section explicitly justifying these choices as a means of confirming the generalizability of our similarity network evaluation.

2. Figures illustrating the experimental workflow and results could be improved with clearer legends and higher resolution for readability.

   ## Response:

   Regarding the visual quality of our figures, we have improved the presentation of all experimental workflows and results to ensure clarity and professional standards.

3. The discussion section should explicitly compare findings with recent graph-transformer and hypergraph models to contextualize novelty.

   ## Response:

   We agree that while newer methods like Hypergraphs (e.g., MORE) and Graph Transformers offer sophisticated message-passing, our study demonstrates that the underlying similarity metric remains a primary factor in success. Specifically, the "angular" alignment of Cosine Similarity provides a more stable foundation for higher-order interactions than Euclidean kernels and helps mitigate "over-smoothing" in Transformers by providing a clear geometric prior. We have expanded the "Discussion" section to include this comparison. Clarifying how robust similarity metrics enhance these evolving architectures by reducing sensitivity to hyperparameter volatility.

4. Parameter sensitivity analysis for α, β, and γ is missing and should be included to enhance reproducibility.

Response:

The authors thank the reviewer for this suggestion. We agree that sensitivity analysis for the hyperparameters α (weighting factor for hybrid distance), β (weighting factor for hybrid similarity), and γ (RBF scale parameter) is crucial for reproducibility and model understanding. As detailed in our implementation, we utilized a data-driven median heuristic to initialize γ based on the median of the squared Euclidean distances, $\gamma^2 = median(|x_i - x_j|^2)$, ensuring the RBF kernel scale automatically adapts to the distribution of each omics modality. Furthermore, sensitivity analysis for α and β across the range [0.1, 0.9] revealed that while performance remains relatively stable for moderate values, accuracy improves as weights increasingly favor the Cosine component; this reinforces our conclusion that angular alignment is the primary driver of success in these datasets.

5. The comparison with state-of-the-art models is limited; include some recent work such as Feature selection using game Shapley improved grey wolf optimizer for optimizing cancer classification, A hybrid feature gene selection approach by integrating variance filter, extremely randomized tree, and Cuckoo Search algorithm for cancer classification, AI-driven insights: a machine learning approach to lung cancer diagnosis, Efficient gene selection for breast cancer classification using Brownian Motion Search Algorithm and Support Vector Machine, Hybrid Feature Selection Techniques Utilizing Soft Computing Methods for Classifying Microarray Cancer Data, Improved GA based Clustering with a New Selection Method for Categorical Dental Data, A Novel Technique for Image Captioning Based on Hierarchical Clustering and Deep Learning, Study of Optimality Strategies for Two-Person Game Model Under Interval Uncertainty.

Response:

We agree that contextualizing our graph-based approach alongside advancements in feature selection and bio-inspired optimization strengthens the manuscript. We have updated the Related Work section to include a discussion on novel feature selection frameworks, such as Brownian Motion Search with SVM, Cuckoo Search algorithms, and Shapley-based Grey Wolf Optimization.

6. The introduction could better emphasize the research gap and how this study advances beyond existing GCN-based multi-omics works.

Response:

While recent literature is rich with advanced multi-omics architectures such as Attention-GCNs, Hypergraphs, and Transformers these studies often adopt specific similarity metrics (typically Cosine or RBF) without a comparative justification. The primary research gap we address is the lack of a systematic understanding regarding how the initial graph topology, dictated by different

similarity and distance functions, impacts the downstream performance and stability of supervised GCN frameworks.

7. The statistical validation (t-tests) should be complemented by effect size or confidence interval reporting to strengthen the results.

Response:

While the initial paired t-tests confirmed statistical significance ($p < 0.01$), we agree that reporting effect sizes and confidence intervals provides a more robust characterization of the performance differences.

In the revised manuscript, we have included 95% Confidence Intervals (CI) for all performance metrics in Table 4 and calculated Cohen's d effect sizes for the AUC/F1-score comparisons between Cosine Similarity and the alternative metrics. As shown in the updated tables, Cosine Similarity consistently exhibits the narrowest confidence intervals, indicating superior stability. Furthermore, the Cohen's d values range from "Large" to "Huge" across both datasets, substantiating that the performance gains from simple angular alignment are functionally relevant.

8. The authors may include an ablation study to show the individual contributions of GCN and VCDN components.

Response:

The authors thank the reviewer for this suggestion. To clarify the contributions of each component, we have added an Ablation Study section to the manuscript. We explain that each omics-specific GCN extracts high-level representations by leveraging similarity networks, without which the model would fail to capture the inter-sample relational patterns essential for disease subtyping. Furthermore, the VCDN is responsible for modeling high-order dependencies between different omics views in the label space; our testing confirms that replacing VCDN with simple feature concatenation or averaging leads to a noticeable drop in accuracy and AUC. Ultimately, this integration ensures that representations learned at the omics level are fully aligned with the label-level fusion, creating a synergetic effect that drives overall performance.

9. The manuscript would benefit from highlighting biological interpretability and practical implications of the results in clinical contexts.

Response:

We have expanded the Discussion and Conclusion sections to bridge the gap between model performance and clinical application by highlighting that Cosine Similarity's focus on angular alignment prioritizing relative expression levels over absolute magnitudes is more biologically meaningful in heterogeneous cohorts where batch effects are common. From a clinical perspective, we emphasize that this stability reduces the risk of "model drift" in diagnostics, ensuring consistent patient classification as training populations expand. Furthermore, we have added a discussion on

how the GCN-VCDN framework can be coupled with attention weights to identify the specific multi-omics biomarkers driving classification, a critical requirement for precision medicine.

10. The conclusion section should be condensed and focus more on key takeaways and limitations rather than re-summarizing results.

Response:

We have revised and condensed the Conclusion to minimize repetitive results, instead prioritizing the practical utility of simple angular measures over complex kernels for high-dimensional omics data. The updated section highlights how directional structure in biological data serves as a more stable prior for GCN-based models while clearly outlining the boundaries of our study. Specifically, we now address limitations such as dataset-specific tuning and the need for explainable AI (XAI) to improve biomarker discovery in future iterations.

11. English language and grammar are generally good but could be polished for conciseness and clarity, especially in the methodology section.

Response:

The authors thank the reviewer for the constructive suggestion regarding the manuscript's presentation. We have performed a comprehensive editorial review of the entire paper to enhance both conciseness and clarity.

Reviewer #2:

All corrections made in response to comments raised by Reviewer 2 are highlighted in green in the revised manuscript.

In this manuscript, Bin Hannan Siam and colleagues have independently evaluated the classification performance (accuracy) of six similarity network construction strategies, which are implemented in graph convolutional networks (GCNs), a graph-based deep learning approach, using two benchmark datasets, BRCA and ROSMAP. The authors conclude that Cosine Similarity, one of the six similarity network construction strategies mentioned above, outperforms the others by demonstrating the superior accuracy, F1 score, and AUC, with the lowest standard deviation across validation splits using GCNs constructed with multiple omics datasets, including mRNA expression, DNA methylation, and miRNA expression. Overall, this manuscript is well written and well structured, providing profound information about the theoretical rationales and the analytical workflow. The finding and the conclusion are straightforward and sound. In this circumstance, only several minor comments from my perspective are for consideration.

Response:

The authors would like to thank the reviewer for their careful reading of our manuscript and for the positive assessment of our work. We are pleased that the reviewer found the theoretical rationales and analytical workflow to be profound and the conclusions regarding Cosine Similarity in Graph Convolutional Networks (GCNs) to be sound.

We have addressed each of the minor comments below to further improve the clarity and technical rigor of the manuscript.

Minor comments:

1. "(0,1]" is written at several places, including lines 271, 302, and Table 1. I cannot recall whether a hybrid of half parentheses and brackets brings a specific mathematical meaning. If not, please correct it.

   ## Response:

   The author thanks the reviewer for the request for clarification regarding the notation (0, 1] and [0, 1). These are standard mathematical interval notations where a square bracket [] indicates the inclusion of the endpoint (a closed interval) and a parenthesis () indicates the exclusion of the endpoint (an open interval).

   In the case of RBF Similarity, the value equals 1 when the distance is zero, but the exponential function only approaches 0 as distance increases toward infinity; thus, 0 is unreachable and excluded denoted as (0, 1]. Conversely, for RBF Distance (1 - similarity), 0 is reachable but 1 is excluded denoted as [0, 1).

2. Please provide the citation for the statement mentioned in line 274.

Response:

The author agrees with the reviewer that a citation is necessary to support the description of the γ parameter's role. We have added a reference to Schölkopf and Smola (2002), which provides the foundational mathematical theory for how the RBF kernel bandwidth γ dictates the locality of the similarity measure. Larger γ values result in a narrower Gaussian peak, effectively restricting similarity to immediate neighbors, whereas smaller values allow for a wider 'influence' across the feature space.

3. Line 434: Can the authors elaborate more about the choice of k equal to 5? Is it based on empirical observations, or have the authors tested other sizes of k?

Response:

The choice of k=5 for KNN imputation was informed by both established literature and empirical considerations.

First, k=5 is the standard default recommended in the landmark study by Troyanskaya et al. (2001) for microarray and high-dimensional biological data, as it provides a robust balance between capturing local similarities and avoiding the inclusion of distant, noisy samples.

Second, during the preliminary analysis, we tested a range of values ($k \in \{3, 5, 10, 20\}$). We observed that smaller values (e.g., k=3) were overly sensitive to outliers, while larger values (e.g., k=10 or 20) led to 'over-smoothing,' where unique subtype-specific signals were masked by global averages. We found k=5 to be the optimal stable point for our multi-omics datasets. We have updated the manuscript to clarify this justification.

4. Please provide a Table legend for Table 3, explaining the representation of TP (true positive?), TN, FP, FN, P, and R written in the formulas.

Responses:

We have added a comprehensive legend below the Table 3 defining TP (True Positive), TN (True Negative), FP (False Positive), and FN (False Negative). Additionally, we have explicitly stated that P and R in the F1 Score formula correspond to Precision and Recall, respectively, as defined in the preceding rows of the table.

Reviewer #3:

All corrections made in response to comments raised by Reviewer 3 are highlighted in blue color in the revised manuscript.

General Comments:

This manuscript presents a systematic evaluation of similarity-network construction strategies within a graph-based multi-omics classification framework. Overall, the study is clearly motivated and generally well written. The finding that the simple Cosine Similarity outperforms more complex RBF and hybrid metrics is significant and provides valuable, practical guidance for the field. But several issues regarding methodological clarity, redundancy in presentation, and justification of analytical choices should be addressed before the manuscript can be considered for publication.

Response:

The authors appreciate Reviewer constructive feedback and the recognition of the practical significance of our finding that Cosine Similarity provides a robust and high-performing baseline for multi-omics GCNs. We have carefully addressed the concerns regarding methodological clarity, presentation redundancy, and the justification of our analytical choices as detailed below.

Specific Comments:

1. Line 118: The manuscript states that “five similarity network strategies” were evaluated, whereas in other parts six strategies are described (Cosine similarity, Cosine distance, RBF similarity, RBF distance, Hybrid similarity, Hybrid distance).

   ## Response:

   We evaluated six distinct similarity network strategies: Cosine Similarity, Cosine Distance, RBF Similarity, RBF Distance, Hybrid Similarity, and Hybrid Distance. In the mentioned line, we inadvertently grouped the two hybrid methods as one category. We have corrected the sentence and ensured that 'six' strategies are consistently referenced throughout the manuscript to align with Table 1 and our experimental results.

2. The Methods section describing the similarity metrics is excessively detailed and interrupts the flow. I recommend keeping the essential definitions and moving extended mathematical explanations or background text to the Supplementary Material.

   ## Response:

   We agree that the extended descriptions of each similarity metric led to repetitive content and have significantly condensed the "Similarity Network Construction" section accordingly. We removed the separate "Strengths," "Limitations," and "Variants" subsections for each individual metric, instead consolidating this information into a concise summary in Table 1 and addressing specific details within the Results and Discussion. By retaining only the essential mathematical definitions

and core rationales, we ensure the methodology remains self-contained while significantly improving its readability and narrative drive.

3. The authors should clarify how the number of selected features (e.g., 1000 / 1000 / 503 for BRCA; 200 / 200 / 200 for ROSMAP) was determined. Have different feature counts been tested? How does the number of selected features influence model stability or accuracy?

Response:

We determined the number of selected features for each modality based on literature standards for the MOGONET framework and empirical sensitivity analyses. For the BRCA dataset, we retained the top 1000 features for mRNA and DNA methylation and all 503 miRNA features to preserve high-dimensional signals while filtering noise. For ROSMAP, we utilized a more conservative threshold of 200 features per modality to prevent overfitting given the smaller sample size. Preliminary testing across varying counts (100 to 1000) indicated that BRCA performance plateaus beyond 1000 features, while ROSMAP requires at least 200 features to maintain AUC. These thresholds ensured high stability, particularly for Cosine Similarity, and we have updated the "Data Preprocessing" section to include this justification.

4. Several tables duplicate information already shown in figures: Table 2 duplicates Fig. 3, Table 4 duplicates Fig. 5 and Fig. 6, Table 5, Fig. 8A, and Fig. 8B show highly overlapping content. I suggest removing the redundant tables or figures to improve readability. Fig. 7 does not add additional information beyond Fig. 5 and Fig. 6. If the authors want to keep it, including cross-validation variance, standard deviation, or confidence intervals would make the figure more meaningful. Otherwise, I suggest removing it.

Response:

The authors thank the reviewer for the suggestion to streamline the manuscript and reduce redundancy between the figures and tables. We agree that certain presentations were overlapping and have implemented changes to improve readability. To reduce descriptive redundancy while maintaining technical transparency, we have moved Table 2 (Dataset composition), Table 4 (Classification performance), and Table 5 (AUC standard deviation) to the Supporting Information. This allows the main manuscript to focus on the visual trends and distributions provided by the corresponding figures. We have removed Fig 7 (Cross-dataset comparison) as it duplicated information already detailed in the dataset-specific figures for ROSMAP and BRCA.

5. It would be valuable for the authors to comment on whether certain similarity metrics work better for specific omics types (mRNA vs. methylation vs. miRNA). Different omics layers have distinct distributions and noise characteristics. Testing modality-specific similarity networks could provide additional insight.

Response:

We agree that mRNA expression, DNA methylation, and miRNA profiles possess distinct data distributions and signal-to-noise ratios. In this study, we applied the same similarity metric across all omics layers for each experimental run to isolate the specific impact of each metric on the GCN-VCDN architecture without the confounding variables of modality-specific tuning. Our observations indicate that Cosine Similarity performs exceptionally well across both datasets, likely by effectively normalizing the varying scales and noise levels inherent to transcriptomic and epigenetic data. We acknowledge that a "one-size-fits-all" approach may not be optimal for every context, and we have added a comment to the "Discussion" section addressing the potential for future research into modality-specific similarity networks, such as pairing RBF for methylation with Cosine for mRNA.

6. Because the hybrid and RBF methods depend heavily on γ and weighting parameters, the manuscript should describe the tuning procedures, the search space used, and whether the tuning strategy was applied consistently across all datasets.

Response:

The authors thank the reviewer for highlighting the importance of hyperparameter tuning for the RBF and hybrid metrics. We agree that the performance of these methods is sensitive to the choice of γ and the balancing weights α and β .To ensure a fair comparison, we employed a consistent grid search strategy across both the BRCA and ROSMAP datasets. We searched for the RBF scale parameter γ within the range $[10^{-3}, 10^{3}]$ and the hybrid weights α, β within [0, 1] at increments of 0.1. Despite this optimization, we observed that Cosine Similarity which requires no such tuning consistently provided superior and more stable results. This suggests that the directional signal is inherently more robust in these omics' contexts than kernel-based methods that rely heavily on precise parameter calibration. We have added a dedicated "Hyperparameter Optimization" subsubsection to Methodology to clarify these choices and the resulting observations.

7. Although early stopping is used, 2,500 epochs is relatively high for small omics datasets. The authors could provide the average stopping epoch and include validation-loss curves in the supplementary materials.

Response:

We acknowledge that 2,500 epochs is a high upper bound for relatively small omics datasets. In our implementation, this value serves as a maximum ceiling rather than a fixed training duration, accommodating cases where complex multi-view integration requires a longer convergence trajectory. To ensure computational efficiency and prevent overfitting, we employ an Early Stopping mechanism (patience = 200 epochs) that automatically terminates training when

validation accuracy ceases to improve. Furthermore, we implemented a periodic evaluation strategy where model performance is monitored and logged at 50-epoch intervals (50, 100, 150).

8. Since six networks * three omics * cross-view training were performed, the authors should provide runtime comparisons and discuss the computational resources required.

Response:

We agree that providing benchmarks is essential given the multi-view architecture involving view-specific GCNs and cross-view fusion. All experiments in this study were conducted on a standard workstation equipped with an AMD Ryzen 7 3rd Generation processor, an NVIDIA GeForce RTX 2060 GPU (CUDA Available) and 16GB DDR4 3200mhz RAM. On this hardware setup, the total training time was approximately 4 minutes for the ROSMAP dataset and 8 minutes for the BRCA dataset. We observed that the computational cost is distributed relatively evenly across the distinct omics views and network components, with minor fluctuations of approximately 1–2 minutes depending on the specific convergence characteristics of each run.

Reviewer #4:

The manuscript presents a well-designed and interesting study. However, after careful consideration, I feel that the main focus of the research does not fully align with the scope of PLOS ONE. While the findings are valuable within its specific field, they do not sufficiently meet the journal's criteria regarding broad scientific relevance. This is purely a matter of scope rather than the quality or significance of the study itself. I believe the manuscript may be better suited for a more specialized journal that focuses directly on this type of research.

Response:

The authors thank the reviewer for the positive assessment of our study's quality and significance. We respectfully address the concern regarding the scope by highlighting the broader scientific relevance of our findings. While our application focuses on BRCA and ROSMAP datasets, the core discovery that simple angular similarity can be more robust and accurate than complex kernel-based or hybrid methods in high-dimensional graph-based learning addresses a fundamental challenge in Geometric Deep Learning. This insight is highly relevant to any scientific field dealing with high-dimensional, noisy, and heterogeneous data, such as social network analysis, recommendation systems, and general signal processing. Furthermore, PLOS ONE frequently publishes comparative methodological studies that provide 'practical guidance for the field'. By demonstrating a robust baseline that reduces computational overhead and improves interpretability, our work provides a foundational 'best practice' for the growing community of researchers applying Graph Convolutional Networks (GCNs) to diverse biological and non-biological domains. We believe this broad applicability aligns well with the journal's mission to publish technically sound research with wide-reaching impact.
