# Supplementary material for: Effects of similarity networks in graph-based multi-omics classification
Source: PLoS One. 2026 Mar 19;21(3):e0344754. doi: 10.1371/journal.pone.0344754 (PMC13001923; doi:10.1371/journal.pone.0344754)
Supplement: Attachment — Submitted filename: Response_to_Reviewers_auresp_2.pdf [file pone.0344754.s010.pdf]

Reviewer Comments

Reviewer #1:

None, the authors have adequately addressed all the comments raised in the previous round of review. I find the revised manuscript acceptable for publication.

Response:

The authors thank the Reviewer for their careful review of our revisions and for the recommendation for publication.

Reviewer #2:

The authors have carefully addressed all my comments. At this stage, I don't have any more comments, so I recommend this manuscript for publication.

Response:

The authors sincerely thank the Reviewer for their time and for the constructive feedback throughout the review process. Also we appreciate the recommendation for the manuscript's publication.

Reviewer #3:

All corrections made in response to comments raised by Reviewer 3 are highlighted in yellow in the revised manuscript.

The authors have carefully considered and adequately addressed my main concerns from the previous round. The manuscript has improved substantially in clarity, organization, and methodological justification. At this stage, I have no major scientific concerns. My remaining comments mainly relate to presentation and redundancy. There is still considerable overlap in the information presented across multiple figures and tables, which affects the readability and conciseness of the manuscript. By implementing some consolidations, the core message—that Cosine Similarity is the most robust and accurate metric —will be supported by a much cleaner and more professional presentation.

Response:

The authors sincerely thank the Reviewer for the positive feedback regarding the improvements in clarity, organization, and methodological justification. We appreciate the reviewer's recognition of our core findings regarding the robustness of Cosine Similarity.

1. In particular, Figure 5 and Figure 6, both figures use three different visual formats (Radar plot, Violin plot, and Heatmap) to present the same set of performance metrics (Accuracy, F1, and AUC). Keep only one sub-figure (for example Figure 5C and 6C) per dataset is recommended.

Response:

We agree that presenting the same metrics (Accuracy, F1, and AUC) across three different visual formats created unnecessary redundancy. Following the reviewer's recommendation, we have streamlined these figures. We have retained the Heatmaps as the primary visualization for both the BRCA and ROSMAP datasets. We removed the Radar plots and Violin plots from the main manuscript and moved it to Supporting Information.

2. Tables 3 and 4 vs. Figures 5 and 6: Tables 3 and 4 provide Mean AUC, SD, and Cohen's d. If the Heatmaps (5C/6C) are kept, consider moving the "Cohen's d" and "95% CI" columns to the text or a supplementary table to avoid repeating the "Mean AUC" and "SD" values cross three different locations (Table, Figure Heatmap, and Supporting Information).

Response:

We agree that the triple-reporting of Mean AUC and SD across tables, heatmaps, and supporting documents hindered the manuscript's flow. As we have selected the Heatmaps (Figures 5 and 6) as our primary visual format for the results, we have moved the Table 3 and Table 4 to the Supporting Information. (Table 3 now appearing as S4 Table and Table 4 as S5 Table.)

3. Figure 7 presents the standard deviation of AUC using both a line plot (A) and a bar chart (B). I recommend keep only Figure 7B (Bar Chart). Ensure the numerical values from S3 Table (AUC standard deviation) are cited in the text so that the visual trend in Figure 7B is sufficient on its own.

Response:

As recommended, we have removed the line plot (7A) and retained only the bar chart (7B). This chart now serves as the updated Figure 7, providing a clean and professional visualization of model stability. We have added the text in the Cross-Validation & Statistical Significance' subsection to precisely reflect the numerical values from S3 Table.
